# Supplementary material for: Splice-Junction-Based Mapping of Alternative Isoforms in the Human Proteome
Source: Cell Rep. Author manuscript; Available in PMC 2020 Jan 15. (PMC6961840; doi:10.1016/j.celrep.2019.11.026)
Supplement: 2 [file NIHMS1546469-supplement-2.zip › DF1/PXD010154/Heart/Heart_76_TTN_ETPPVEVPGGEK.pdf]

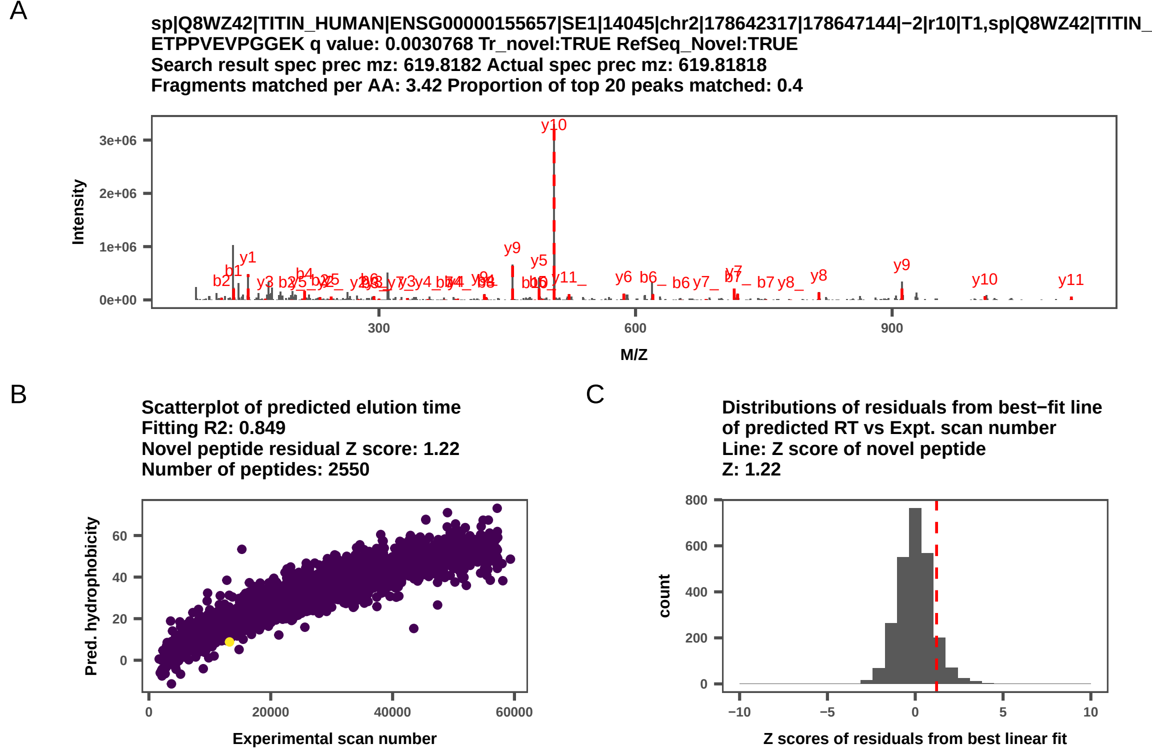
A
sp|Q8WZ42|TITIN_HUMAN|ENSG00000155657|SE1|14045|chr2|178642317|178647144|−2|r10|T1,sp|Q8WZ42|TITIN_
ETPPVEVPGGEK q value: 0.0030768 Tr_novel:TRUE RefSeq_Novel:TRUE
Search result spec prec mz: 619.8182 Actual spec prec mz: 619.81818
Fragments matched per AA: 3.42 Proportion of top 20 peaks matched: 0.4
Intensity
3e+06
2e+06
1e+06
0e+00
y10
y9
y5
y1
b1
b2
y6
y9
y10
y11
y8
M/Z
300
600
900
B
Scatterplot of predicted elution time
Fitting R2: 0.849
Novel peptide residual Z score: 1.22
Number of peptides: 2550
Pred. hydrophobicity
60
40
20
0
0
20000
40000
60000
Experimental scan number
C
Distributions of residuals from best−fit line
of predicted RT vs Expt. scan number
Line: Z score of novel peptide
Z: 1.22
count
800
600
400
200
0
−10
−5
0
5
10
Z scores of residuals from best linear fit
